# A zebrafish model of glucocorticoid resistance shows serotonergic modulation of the stress response

**DOI:** 10.3389/fnbeh.2012.00068

**Published:** 2012-10-11

**Authors:** Brian B. Griffiths, Peter J. Schoonheim, Limor Ziv, Lisa Voelker, Herwig Baier, Ethan Gahtan

**Affiliations:** ^1^Department of Psychology, Humboldt State UniversityArcata, CA, USA; ^2^Program in Neuroscience, Department of Physiology, University of California, San FranciscoSan Francisco, CA, USA; ^3^Institute of Biology, Leiden UniversityLeiden, Netherlands; ^4^Cancer Research Center, Sheba Medical CenterTel Hashomer, Israel

**Keywords:** glucocorticoid receptors, zebrafish, SSRI, stress, depression, anxiety

## Abstract

One function of glucocorticoids is to restore homeostasis after an acute stress response by providing negative feedback to stress circuits in the brain. Loss of this negative feedback leads to elevated physiological stress and may contribute to depression, anxiety, and post-traumatic stress disorder. We investigated the early, developmental effects of glucocorticoid signaling deficits on stress physiology and related behaviors using a mutant zebrafish, *gr*^*s357*^, with non-functional glucocorticoid receptors (GRs). These mutants are morphologically inconspicuous and adult-viable. A previous study of adult *gr*^*s357*^ mutants showed loss of glucocorticoid-mediated negative feedback and elevated physiological and behavioral stress markers. Already at 5 days post-fertilization, mutant larvae had elevated whole body cortisol, increased expression of pro-opiomelanocortin (POMC), the precursor of adrenocorticotropic hormone (ACTH), and failed to show normal suppression of stress markers after dexamethasone treatment. Mutant larvae had larger auditory-evoked startle responses compared to wildtype sibling controls (*gr*^*wt*^), despite having lower spontaneous activity levels. Fluoxetine (Prozac) treatment in mutants decreased startle responding and increased spontaneous activity, making them behaviorally similar to wildtype. This result mirrors known effects of selective serotonin reuptake inhibitors (SSRIs) in modifying glucocorticoid signaling and alleviating stress disorders in human patients. Our results suggest that larval *gr*^*s357*^ zebrafish can be used to study behavioral, physiological, and molecular aspects of stress disorders. Most importantly, interactions between glucocorticoid and serotonin signaling appear to be highly conserved among vertebrates, suggesting deep homologies at the neural circuit level and opening up new avenues for research into psychiatric conditions.

## Introduction

**Figure 1:**
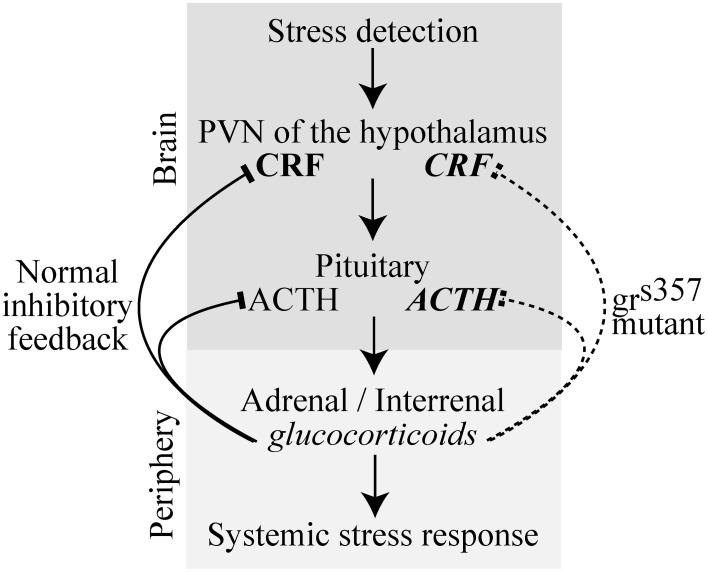
**A simplified schematic of the vertebrate HPA stress pathway in normal and *gr*^*s357*^ mutant zebrafish in which the glucocorticoid receptor is non-functional.** Lines with arrowheads indicate feed forward excitation and lines with blocked heads indicate feedback inhibition. Regular font signifies neural structures and italicized font signifies diffusible signaling molecules. Glucocorticoid feedback from the periphery to the brain normally inhibits further brain stress activity by transcriptional suppression of genes including those encoding CRH and ACTH (left, solid lines). In *gr*^*s357*^ mutant zebrafish, the glucocorticoid receptor is non-functional, preventing negative feedback (right, dashed line), and leading to prolonged release and higher levels of stress intermediaries (bold font). Abbreviations: CRH, Corticotropin releasing hormone; ACTH, Adrenocorticotropic hormone; HPA, Hypothalamic-pituitary-adrenal axis; PVN, Paraventricular nucleus.

Glucocorticoids serve a homeostatic function in acute stress responses by providing negative feedback to brain stress circuits. Activated glucocorticoid receptors (GRs) bind to DNA and regulate the transcription of many genes, including (but not restricted to) components of the hypothalamo-pituitary-adrenal axis. Combined, these expression changes effectively terminate the stress response (Figure 1), but they also have a plethora of other effects on behavior and physiology.

Several neural mechanisms may underlie stress effects on mood and behavior (Howell et al., 2011). Cortisol and Corticotropin Releasing Hormone (CRH) acutely modify limbic system and prefrontal cortex neurons involved in regulation of affect (Meng et al., 2011). Repeated stress may induce long-term neural adaptations, including down regulation of GR expression (Howell et al., 2011), which has been linked to depressive behaviors in rodent models (Zouh et al., 2011). Excessive cortisol in early life can permanently suppress the GR gene by epigenetic modification, leading to elevated stress in adulthood (Wilkinson and Goodyer, 2011).

Stress also contributes to serotonin dysregulation, a hallmark of depression (Anacker et al., 2011). CRH activates a subpopulation of serotonergic neurons in the raphe nuclei, and recruitment of this pathway mediates anxiety-like behavior in rats (Meloni et al., 2008). Chronic stress decreases serotonin receptor expression (Kieran et al., 2010) and serotonin binding in the limbic system and cortex, and lowers behavioral responses to serotonin (Bush et al., 2003). Serotonin also influences hypothalamic stress pathways (Locatelli et al., 2010). Selective serotonin reuptake inhibitors (SSRIs) increase GR expression (Erdeljan et al., 2001), potentially compensating for stress-induced downregulation, and they normalize stress-induced behavioral deficits in animal models (Leventopoulos et al., 2009).

Zebrafish are a promising model for studying human stress-related disorders because of homologies in key genetic, physiological, and behavioral features of stress regulation (Egan et al., 2009; Schaaf et al., 2009; Löhr and Hammerschmidt, 2011; Piato et al., 2011; Ziv et al., 2012). Zebrafish show mature physiological stress reactivity, including GR-mediated negative feedback, by 4 dpf (Alderman and Bernier, 2009; Alsop and Vijayan, 2009; Machluf et al., 2011), allowing stress dynamics to be studied in the experimentally tractable fish larvae. Their small size and aquatic environment allow the monitoring of single-animal behavioral in microtiter format (96-well plates, or similar), a feature that makes them attractive for high-throughput whole-animal *in vivo* screens of small compound libraries (Rihel et al., 2010).

**Figure 2:**
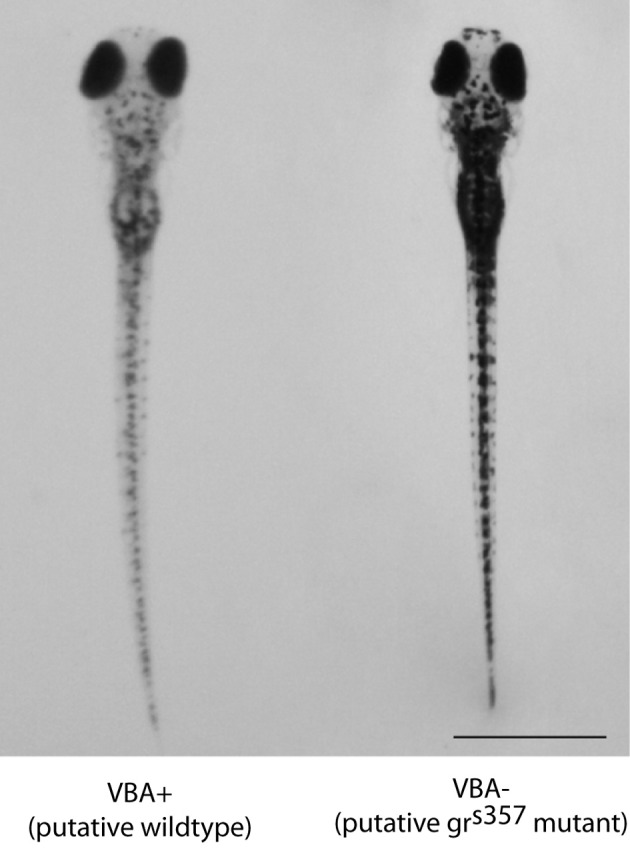
**Representative photographs of a VBA+ (left) and VBA− (right) larva from the same clutch after exposure to the VBA stimulus on day 5 post-fertilization.** The dark fish were reproducibly genotyped and shown to be homozygous *gr*^*s357*^ mutants. Non-dark siblings were either homozygous wildtype or heterozygotes. Scale bar = 1 mm.

We investigated stress behavior and physiology in mutant zebrafish larvae with dysfunctional GRs (*gr*^*s357*^). A previous study showed that the *s357* mutation is a single nucleotide substitution at position 443, a region essential for DNA binding. The mutant GR can bind cortisol and translocate to the nucleus, but shows no transcriptional activity (Ziv et al., 2012). The mutant was identified on the basis of impaired visual background adaptation (VBA; Muto et al., 2005), a GR-dependent neuroendocrine response that causes skin pigment cells to contract under bright illumination (Kramer et al., 2001). *gr*^*s357*^ larvae appear darker than *gr*^*wt*^ in the VBA assay (Figure 2) showed reduced swimming activity (Muto et al., 2005). *gr*^*s357*^ mutants are viable, but adults show elevated behavioral and physiological stress responses. Upon transfer to a novel tank, which is a mild stressor, adult mutants show a longer duration immobilization response, reduced exploration, and they do not habituate after repeated novel tank exposure trials. Fluoxetine or exposure to other zebrafish normalizes these responses in mutants, strengthening the conclusion that the GR mutation results in a depression-like phenotype (Ziv et al., 2012).

Here we demonstrate that GR-mediated gene regulation and stress reactivity are measurably altered in *gr*^*s357*^ mutant larvae already shortly after hatching, when they begin to swim and search for food. Addition of the SSRI fluoxetine to the tank water was able to modify these parameters in both mutants and wildtype larvae. These findings show a contribution of the serotonin signaling system to naïve (unconditioned) stress response and extend the phylogenetic conservation of stress mechanisms to brain circuitry and behavior.

## Materials and methods

### Animals

Adult *gr*^*s357*^ zebrafish were generated and maintained as described (Muto et al., 2005). Adult breeding pairs were confirmed heterozygous *gr*^*s357*^ mutant carriers by genotyping of fin clip samples (^*^), and by the distribution of VBA—phenotype in larval clutches. Adults and larvae were housed according to standard laboratory conditions (Matthews et al., 2002). A 14/10 light/dark cycle was maintained to entrain circadian rhythms. All larvae used in behavioral experiments were siblings from the same parents. All procedures were approved by an IACUC review committees at Humboldt State University and the University of California, San Francisco, CA.

### Sorting *gr*^*s357*^ mutants by VBA

Clutches were run in the VBA assay on day 5 or 6 post-fertilization to sort mutants from wildtype larvae prior to experimentation. Larvae were placed in 60 mm plastic petri dishes, up to 40 per dish, in standard zebrafish water, and exposed to 20 min darkness by placement in an opaque plastic box, and were then transferred onto a white background under bright, whole-field illumination (using a 30 W fluorescent lamp mounted 18 inches above the dish). These conditions reliably trigger VBA responses in normal zebrafish larvae (Muto et al., 2005; Hatamoto and Shingyoji, 2008).

### Drug treatments

Larvae treated with fluoxetine hydrochloride (Sigma) were incubated in a 4.6 μM fluoxetine solution for the 24 h immediately prior to behavioral testing (starting on day 5 post-fertilization). Fluoxetine was dissolved in regular larval water and pH'd to 7.2. This concentration and timing of exposure was selected on the basis of a previously published study (Airhart et al., 2007). After incubation larvae were transferred using a 2 ml plastic pipette from fluoxetine into a 60 mm rinse dish containing regular egg water, and immediately transferred again into behavior recording wells containing regular egg water. Control larvae received equal number and timing of transfers but were not exposed to fluoxetine. Larvae treated with betamethasone 17-valerate (a synthetic steroid and cortisol analog; Sigma) were incubated in a 25 or 30 uM solution for 24 h prior to euthanizing and processing for *in situ* hybridization (ISH). Betamethasone was dissolved in 100% ethanol to make a 1000× stock solution, and then diluted in regular egg water for bath application to larvae. Control larvae were incubated in the same ethanol concentration without Betamethasone added.

### Behavioral assays

Larval behavior was recorded with digital video cameras (Pixelink PLA-740 for spontaneous activity recordings, PhotonFocus MV1-1312 for startle swims) and images were stored on a computer for offline analysis. The behavior apparatus was housed inside a sound-attenuating cabinet. Infrared (IR) illumination was used for image capture and the recording area was maintained in darkness. Larvae were individually transferred using a 2 ml plastic pipette into wells (7 mm diameter) of a clear plastic 24 well tissue culture plate (Fisher Scientific) containing 1 ml of larval medium per well. The plate was positioned on a glass shelf and a white, light diffusing plastic sheet was placed on top of it. The IR illuminator (Raytec Systems Inc) was positioned above the plate, and cameras below, providing high-contrast, shadow-free images. Up to 4 plates (96 larvae) were recorded simultaneously. A computer controlled image acquisition and presentation of sensory stimuli through a USB input-output board (Labjack U-12) run by a control program written specifically for each experiment. Temperature in the recording area was automatically logged at 10 min intervals and maintained at 27 ± 2°C.

The spontaneous activity assay was run at 5–6 dpf, immediately after VBA sorting of mutants. Activity was recorded in darkness to remove visually mediated effects and allow assessment of circadian entrainment. Images were acquired at a rate of 0.5 Hz for 24 h, beginning at zeitgeber time (ZT) 20 h. Two spontaneous activity experiments were conducted using separate clutches. The first compared activity level and circadian rhythm in *gr*^*s357*^ mutants and controls (*N* = 48 per group) across a 24 h recording period. In the second experiment, half of the larvae in each genotype group (*N* = 24 per group) were treated with fluoxetine (4.6 μM for 24 h prior to testing) and activity was recorded for 8 h (from CT 13–21 h). Automated computer analysis of the images determined whether a larva had moved from one video frame to the next (a 2 s interval) for each well across the entire video. There were 1800 video frames per hour, and results were expressed as the percentage of time the larva was in motion per hour (number of frames with movements/1800).

Auditory startle was evoked using a mechanical tapping that produced approximately 75 decibels at the recording plate. This stimulus reliably produced a “startle response” consisting of a C-bend within 200 ms followed by a <1 s bout of rapid swimming. Movement was video recorded at 100 frames per second for 1 s following the tap. Swimming distance and group response rate were analyzed on each trial. One block of trials consisted of five consecutive tap trials with 2 s between taps (10 s total). Five consecutive trial blocks were presented, with 30 min between blocks, resulting in 25 trials total over 150 min. Four groups of larvae were tested simultaneously: untreated *gr*^*s357*^, untreated wildtype, fluoxetine treated *gr*^*s357*^, and fluoxetine treated wildtype. All were siblings from the same clutch. The data were analyzed using repeated measures ANOVAs with trial or block number as the within subjects factors and genotype or drug treatment as the between subjects factors. Movement distance and latency measurements were done by an automated motion tracking computer program, custom written in MySQL for this study. Group response rate, defined as the percentage of each group that met a minimum response criteria of >1 mm of movement within 500 ms post-stimulus, was calculated for each trial. Startle habituation was measured as the statistical effect of trial number, for short-term habituation, or block number, for longer term habituation. Data for a larva was included in the analysis only if the larva met the response criteria (>1 mm movement within 500 ms post-stimulus) on the first trial of the first block. This resulted in removal of 7 larvae each from the untreated *gr*^*s357*^ and wildtype groups, and 10 larvae each from the two fluoxetine treated groups.

### Whole-body cortisol assay

After behavioral recordings the larvae were collected by group, euthanized by overexposure to an anesthetic solution (10 min bath application of 0.1% Tricaine methanesulfonate (Sigma), pH'd to 7.0), and then homogenized with a rounded glass rod inside 1.5 ml Eppendorf tubes. Tubes were spun for 5 min in a microfuge and 50 μl of supernatant was taken for the ELISA assay. Cortisol was measured with a human salivary cortisol ELISA kit (Salimetrics kit 1-3002), following a procedure reported for whole body cortisol measurement in adult zebrafish (Egan et al., 2009). Twenty-four larvae from each group (the same larvae used in behavioral experiments that tested fluoxetine effects) were homogenized together on day 7 post-fertilization in equal volumes of phosphate-buffered saline (PBS) and then split into duplicate samples for testing. The absorbance values obtained were then averaged across the two duplicate samples for each group. Individual larvae could not be tested because of their small size, so inferential statistical tests on cortisol measurements were not feasible.

#### POMC in situ hybridization

The pro-opiomelanocortin (*pomc*) antisense probe was obtained from Deborah Kurrasch (UC Alberta, Calgary). ISH was performed in 5 day old larvae according to a previously reported protocol (Kurrasch et al., 2009). Larvae were fixed in 4% paraformaldehyde in PBS at 4°C for 12 h then stored in 100% methanol at −20°C. Larvae were rehydrated in PBS-Tween (PBS; 0.1% Tween-20), incubated in proteinase K (20 μg/ml; 8 min), refixed in 4% paraformaldehyde for 20 min, prehybridized (65–70°C; 1 h), and hybridized (65–70°C, overnight) with the *pomc* probe. Embryos were washed, blocked in 5% sheep serum, incubated in α-digoxigenin (DIG) antibody overnight at 4°C, washed, and incubated with p-nitroblue tetrazolium chloride/5-bromo-4-chloro-3-indolyl phosphate (NBT/BCIP) in staining buffer (100 mM NaCl, 50 mM MgCl2, 100 mM Tris-HCl, 0.1% Tween-20). The reaction was stopped after approximately 3 h by rinsing with PBSTw, and larvae were then placed in 90% glycerol for clearing.

### Data analysis

Videos of larval movements were quantified using ImageJ (Abramoff et al., 2004). Each video frame was subtracted from a reference image that contained no larvae but was otherwise identical, generating a new video in which only the larvae are visible. The position of each larva on each frame was measured as the XY coordinates of the center of mass the larva's image on each frame. XY coordinates were then imported into a MySQL database (Oracle) for further processing. To distinguish the activity pattern of each larva, coordinate pairs were sorted by the area occupied by each well within the 96-well image. Another script in the database program calculated the distance traveled for each larva, latency of movement onset, and percentage of time in motion per hour and overall. Processed data were exported to PASW to conduct inferential statistical tests. Circadian activity rhythm was analyzed using a repeated measures ANOVA with time (ZT) as the within subjects factor and genotype as the between subjects factors. Startle responses were also analyzed with repeated measures ANOVA, with trial or block number as the within subjects factor, and genotype as the between subjects factor. The effects of Fluoxetine treatment were examined for each genotype in separate analyses. *T*-tests were used to measure group differences in overall spontaneous activity, startle response rate, and pigmentation. The Greenhouse-Geisser correction against unequal variances was applied in every analysis, and reported degrees of freedom were rounded to the nearest whole number. Effect sizes were always reported.

## Results

### Mutant larvae can be sorted by VBA

Clutches from heterozygous mutant parents were consistently sortable on the basis of the VBA test on day 5 post-fertilization. The VBA defect is a reliable phenotypic marker of the homozygous mutant genotype (Ziv et al., 2012). Analysis of two separate clutches from the same identified heterozygous parents showed the expected ratio (1:4) of VBA− and VBA+ larvae. Of 194 larvae, 47 were manually sorted into the dark pigmentation group (VBA−, presumed mutants) and 147 into the light pigmentation group (VBA+), which is consistent with the predicted frequency distribution [χ^2^_(1, *N* = 194)_ = 0.06, *p* = 0.80]. Skin darkness on the dorsal surface of the body was also significantly different between the manually sorted VBA− and VBA+ groups [average 8-bit gray scale value = 82.91 ± 13.31 and 61.45 ± 10.04 for VBA+ and VBA− groups, respectively; *t*_(65)_ = 7.459, *p* < 0.001, *d* = 1.82]. Figure 2 shows photographs of a representative larva from each group, taken immediately after VBA stimulus presentation.

### Physiological stress markers are misregulated in *gr*^*s357*^ mutant larvae

**Figure 3:**
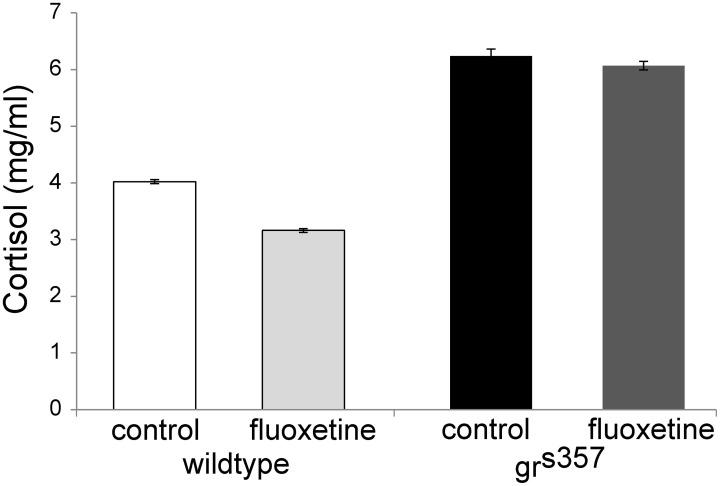
**Whole body cortisol measurements in 7 day old zebrafish larvae.** Cortisol measurements in 7 day old *gr*^*s357*^ mutants and wildtype siblings with and without fluoxetine treatment. Each group consisted of 24 larvae processed together, split into two duplicate samples, and tested concurrently. Values shown are transformed from ELISA absorbance measurements. Error bars are SEMs of the two duplicate samples per group.

Whole body cortisol was measured in 4 groups: *gr*^*s357*^ mutants and wildtype, with and without fluoxetine treatment. Untreated mutants had the highest levels of whole body cortisol (6.07 ± 0.03 mg/ml), approximately 50% greater than that measured for untreated wildtype larvae (4.02 ± 0.03 mg/ml). In both mutants and controls, fluoxetine treatment was associated with slightly lower cortisol levels (Figure 3).

**Figure 4:**
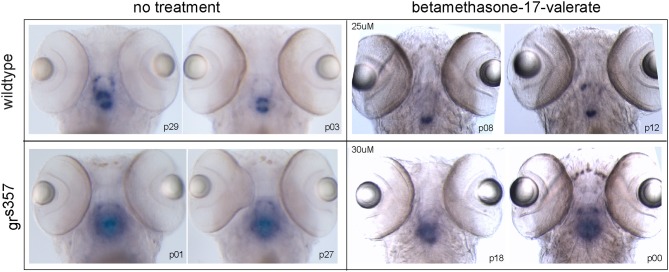
**RNA *in situ* hybridization for *pomc* transcript in 5 day old larvae.** Results are presented from a 2 × 2 experimental design testing the effects of genotype (rows) and betamethasone 17-valerate treatment (columns) on *pomc* expression. Two representative larvae per group are shown (*N* = 5 per group). *pomc* reactivity is localized to the pituitary region in all groups, but in *gr*^*s357*^ larvae expression appeared stronger and showed no suppression after treatment with betamethasone 17-valerate.

Expression of *pomc* RNA was strongest in the region of the pituitary in all 25 larvae examined. The area of the brain showing *pomc* reactivity was consistently larger in *gr*^*s357*^ than in wildtype larvae. Betamethasone 17-valerate, a synthetic glucocorticoid, suppressed *pomc* transcript in wildtype larvae as expected, but did not appear to suppress *pomc* in *gr*^*s357*^ larvae. Figure 4 shows *pomc* labeling in two larvae from each group (*N* = 5 per group). Labeling was absent in a control group in which the *pomc* sense probe was used (not shown).

### *gr*^*s357*^ mutant spontaneous locomotor activity is depressed, but restored by fluoxetine

**Figure 5:**
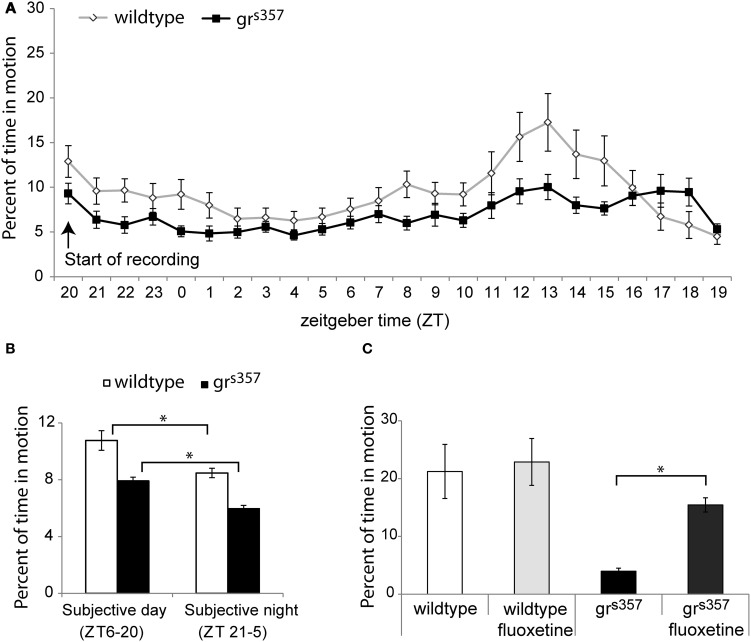
**Spontaneous activity in *gr*^*s357*^ and wildtype larvae. (A)** Average hourly activity (±standard error) across a 24 h spontaneous activity recording (*N* = 48 per group). Both groups showed a circadian activity rhythm with a peak at ZT13, but wildtype larvae had a higher overall activity level. **(B)** Activity as a function of subjective light cycle phase. Both groups had significantly higher activity during the subjective light phase. **(C)** Fluoxetine effects on spontaneous swimming activity (*N* = 24 per group). Fluoxetine increased activity in *gr*^*s357*^ but had no effect in the wildtype group. ^*^P < 0.5.

In both spontaneous activity experiments, untreated *gr*^*s357*^ larvae had significantly lower spontaneous swimming activity than wildtype controls (Figure 5). Average percent of time spent in motion across the 24 h observation was 7.0 ± 1.7% for mutants and 9.5 ± 3.3% for controls [*F*_(1, 65)_ = 5.21, *p* = 0.026, η^2^_*p*_ = 0.07], and across the 8 h observation activity values were 3.95 ± 2.2% and 21.2 ± 18.7% for untreated mutants and siblings, respectively [*t*_(30)_ = 3.7, *p* = 0.001, *d* = 1.30]. Despite lower activity levels, *gr*^*s357*^ showed evidence of a circadian activity rhythm with the same phase as the wildtype group, with peak activity occurring at ZT 13 h (Figures 5A,B).

There were significant effects of fluoxetine treatment on spontaneous activity in the 8 h observation [*F*_(3, 60)_ = 7.3, *p* < 0.001, η^2^_*p*_ = 0.21]. Spontaneous activity was lowest in untreated *gr*^*s357*^ larvae (3.96 ± 2.2%), and was significantly different from activity levels in wildtype controls [21.2 ± 18.7%; *t*_(15, 41)_ = 3.7, *p* = 0.002, *d* = 1.30] and in fluoxetine treated mutants [15.4 ± 4.9%; *t*_(20, 67)_ = 8.5, *p* < 0.001, *d* = 3.01]. Fluoxetine had no effect on spontaneous activity among wildtype larvae [*t*_(30)_ = 0.27, *p* = 0.79, *d* = 0.09; Figure 5C].

### *gr*^*s357*^ mutant startle responses are increased, but normalized by fluoxetine

**Figure 6:**
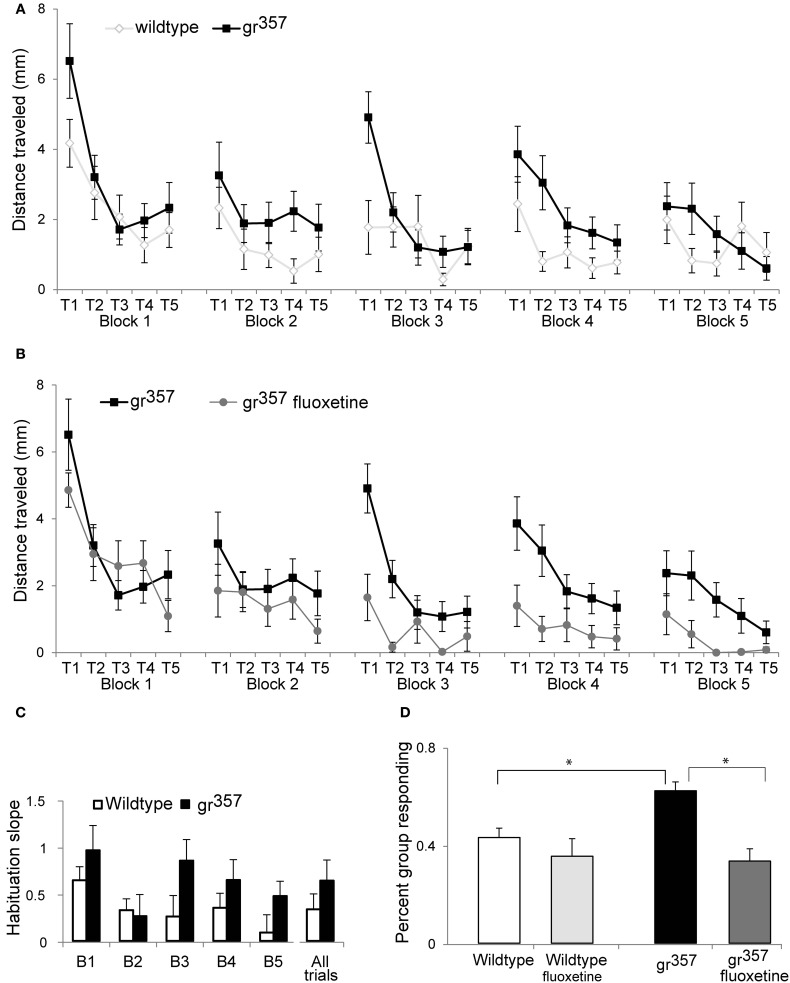
**Startle responses in wildtype (*N* = 17), untreated *gr*^*s357*^ (*N* = 17), and fluoxetine treated *gr*^*s357*^ larvae (*N* = 14). (A)** A comparison of startle magnitude (±SEM) in untreated wildtype and *gr*^*s357*^ larvae. Mutants had significantly larger responses than wildtype across all trials. Habituation was evident both within blocks and across blocks. **(B)** A comparison of startle magnitude in mutant larvae with and without fluoxetine treatment. Fluoxetine resulted in significantly lower startle responses in mutants, but the groups did not differ on measures of habituation. **(C)** Habituation within block shown as the slope of the regression line through the 5 trails on each block, and as an average of all 25 startle trials. Mutants had steeper habituation slopes (more habituation) in 4 out of 5 blocks, and across all 25 trials, reflecting the larger startle responses of mutants on the earliest trials of each block. This graph illustrates a trend in the data, but effect of genotype on habituation was not statistically significant. **(D)** Percent of group responding across all 25 trials. Untreated mutants responded significantly more frequently than wildtype groups or fluoxetine treated mutants (^*^P < 0.05).

The *gr*^*s357*^ group had significantly elevated startle responses (Figure 6). As a group, mutants had larger startle swims on 20 out of 25 trials. Average distance traveled in the 1 s following the startle stimulus was 2.3 ± 2.5 mm for mutants compared to 1.5 ± 2.2 mm for controls [*F*_(1, 32)_ = 6.05, *p* = 0.02, η^2^_*p*_ = 0.16]. There were significant main effects of trial number [*F*_(2, 75)_ = 22.0, *p* < 0.001, η^2^_*p*_ = 0.41] and block number [*F*_(3, 110)_ = 5.2, *p* = 0.001, η^2^_*p*_ = 0.14], reflecting declining response magnitudes across trials and blocks (i.e., habituation). There were no significant interaction effects with genotype, implying no differences in habituation between mutants and controls. However, because *gr*^*s357*^ had larger responses on the first two trials of each block, the overall rate of habituation within blocks was numerically greater for *gr*^*s357*^ than for wildtype controls (Figures 6A,C).

Fluoxetine treatment resulted in significantly smaller startle responses in mutants [*F*_(1, 29)_ = 10.2, *p* = 0.003, η^2^_*p*_ = 0.26; Figures 6B,D] but had no effect in wildtype controls [*F*_(1, 29)_ = 0.4, *p* = 0.517, η^2^_*p*_ = 0.015; data not shown]. There were significant main effects of trial and block in mutants [*F*_(2, 67)_ = 198.5, *p* < 0.001, η^2^_*p*_ = 0.40 for trials, *F*_(3, 95)_ = 13.05, *p* < 0.001, η^2^_*p*_ = 0.31 for blocks] and in wildtype controls [*F*_(3, 85_ = 8.9, *p* < 0.001, η^2^_*p*_ = 0.23 for trials, *F*_(3, 97)_ = 18.1, *p* < 0.001, η^2^_*p*_ = 0.38 for blocks], but no interaction effects between fluoxetine and trials or blocks. Therefore, fluoxetine did not alter the dynamics of habituation with repeated elicitation of startle (Figure 6B). Response rate was higher among untreated mutants versus wildtype controls in 22 out of the 25 trials run. The percent of the group responding across all 25 trials was 62.1 ± 17.7% for untreated *gr*^*s357*^ larvae compared to 43.1 ± 19.0% for untreated controls, and this difference was significant [*t*_(48)_ = 3.66, *p* = 0.001, *d* = 1.03; Figure 6D]. Untreated mutants also had significantly higher response rate than fluoxetine treated mutants [*t*_(48)_ = 4.65, *p* < 0.001, *d* = 1.32], but fluoxetine did not affect startle response rate in wildtype larvae.

## Discussion

Physiological and behavioral indices of stress were both found to be elevated in *gr*^*s357*^ mutant zebrafish larvae possessing a non-functional GR. The hyper-stress phenotype of *gr*^*s357*^ likely results from the loss of negative feedback to brain stress circuits that is normally mediated by glucocorticoid signaling in the hypothalamus (Figure 1). The failure of *gr*^*s357*^ mutant larvae to respond to treatment with betamethasone (a synthetic glucocorticoid) by inhibiting *pomc* expression, as wildtype larvae did, is the most direct evidence presented here that negative feedback is missing in the mutant. Increased baseline levels of pituitary *pomc* and whole body cortisol also support this mechanism. These effects on physiological stress markers in *gr*^*s357*^ larvae are quite similar to those seen in the adult mutants (Ziv et al., 2012) and are remarkable given the young age of the fish. Sequence analysis localized the mutation to a critical GR DNA binding region in the second zinc finger domain and transcriptional assays found the mutant receptor completely unable to mediate gene regulatory responses to exogenous cortisol (Ziv et al., 2012). The VBA-deficient phenotype of the mutant, which led to its original discovery (Muto et al., 2005) might be related to an upregulation of alpha-melanocyte stimulating hormone, which is encoded by the POMC precursor. The failure to concentrate melanin pigment is thus consistent with a lack of GR-mediated negative feedback on *pomc* expression.

The *gr*^*s357*^ mutant larvae had larger behavioral startle responses than wildtype despite having lower levels of spontaneous locomotor activity. Therefore, the startle analysis may underestimate the difference between mutants and wildtype larvae; startle measurements did not account for baseline activity levels, and mutants exhibited a greater baseline-to-startle activity difference. This result also suggests that the spontaneous activity deficit in mutants is not due to an inability to move as quickly as wildtype larvae. Lower spontaneous activity in the mutant was observed in two separate experiments presented here, both of which extend a previous, semi-quantitative finding (Muto et al., 2005). Zebrafish are known to freeze under certain stress conditions, including being placed in novel environments (Egan et al., 2009). Lower spontaneous activity in *gr*^*s357*^ may therefore be a behavioral expression of stress deriving from internal physiology.

Although the differences in startle and spontaneous activity between *gr*^*s357*^ and wildtype larvae were statistically significant, they were moderate in size. The fact the *gr*^*s357*^ zebrafish are adult-viable also implies constraints on the severity of their behavioral impairments as larvae, since very severe impairments would be lethal. Considering the many genes, physiological systems, and developmental processes that are influenced by glucocorticoid signaling, it seems surprising that GR knockout in zebrafish does not result in a more extreme behavioral phenotype. However, this result is consistent with reports on targeted GR gene knockout mice. In one study, GR knockout mice that survived to adulthood were behaviorally normal despite elevated levels of adrenocorticotropic hormone (ACTH) and cortisol (Oitzl et al., 1997). In another study, GR knockout in the forebrain in mice caused moderate depression-like symptoms in the open field and elevated plus maze tests (Wei et al., 2004). One reason that stress behavior may be only moderately increased by loss of GR signaling in *gr*^*s357*^ is that behavioral responses to corticosteroids are partly mediated by mineralocorticoid receptors. In mice, mineralocorticoid receptors help mediate stress effects on locomotor behavior, aggression, and memory formation (Groeneweg et al., 2011). Mineralocorticoid receptors are presumed to functional normally in *gr*^*s357*^ larvae and are expressed at normal levels (Ziv et al., 2012).

Another reason that the health and behavior deficits of *gr*^*s357*^ mutants are not more severe may be that some GR function is preserved in spite of the total loss of DNA binding and transcriptional effects. Activated GRs are known to interact directly with several enzymes and proteins, including other transcription factors, indicating a diversity of glucocorticoid signaling mechanisms (Revollo and Cidlowski, 2009). These non-transcriptional effects of the GR include interactions with proteins such as MAPK that are known to be involved in the regulation of behavioral stress responses (Gutièrrez-Mecinas et al., 2011). It is especially of interest to know whether fluoxetine's effects on *gr*^*s357*^ mutant behavior in this study, including normalization of spontaneous activity levels and startle responses, are mediated by non-transcriptional effects of the GR. Furthermore, any changes in the expression levels of the GR due to a loss of its transcriptional activity could modify these non-transcriptional actions. Since the *gr*^*s357*^ mutation selectively disrupts the DNA-binding domain of the GR protein (Ziv et al., 2012), it is possible that some or all of the GR protein-protein interactions are intact, but studies that directly examine the targets of those interactions in mutant zebrafish are required to confirm that possibility.

Interactions between glucocorticoid and serotonin signaling are strongly implicated in human stress disorders, both in their pathogenesis and treatment (Anacker et al., 2011). One current hypothesis is that the therapeutic effects of serotonergic antidepressants depend on the drugs' ability to decrease HPA activity and restore normal HPA responsiveness (as measured by the dexamethasone suppression test; Knorr et al., 2009). Fluoxetine had opposing effects in *gr*^*s357*^ mutants, increasing spontaneous activity but decreasing startle activity. In both cases, however, fluoxetine treatment brought mutant performance closer to wildtype values. This “normalizing” effect of fluoxetine in *gr*^*s357*^ mutants may resemble therapeutic effects in humans, but the mechanisms are likely to be different.

In humans, SSRI's are proposed to upregulate GR expression, thereby facilitating negative feedback effects of cortisol in the brain (Anacker et al., 2011). In contrast, upregulation of a non-functional GR in *gr*^*s357*^ should not produce any effect. One explanation may be that fluoxetine also influences mineralocorticoid signaling in *gr*^*s357*^, with effects on the brain and behavior (Hlavacova et al., 2010). Fluoxetine had no effects on wildtype behavior in the present study. A previous study that found the same concentration and timing of fluoxetine exposure in zebrafish larvae did depress spontaneous activity, though it had no effect on heart rate or pectoral fin movements (Airhart et al., 2007). This discrepancy may result from the different methods used to measure spontaneous activity.

GR mutation occurs rarely in humans, and can result in a syndrome known as primary glucocorticoid resistance (Ruiz et al., 2001; Bouligand et al., 2010). Patients may be asymptomatic or affected with conditions including fatigue, hypertension, and hirsutism (Trebble et al., 2010). Since these are all heterozygous GR mutations, the range of symptoms may relate to different mutations and degrees of haploinsufficiency. Two consistent findings in glucocorticoid resistance in humans are elevated stress hormones and an impaired dexamethasone suppression test, both of which imply a loss of GR-mediated negative feedback in the brain (Kolber et al., 2008; van Rossum and van den Akker, 2011). Interestingly, glucocorticoid resistance in humans, whether caused by GR mutation or other mechanisms, is not consistently associated with depression or anxiety, while GR hypersensitivity is associated with depression (Anacker et al., 2011).

Zebrafish larvae are increasingly seen as a useful model in which to study principles of stress and stress-related disorders. Homologies between human and zebrafish systems include not only physiological mechanisms but also adaptive functions. For example, both humans (Heinrichs et al., 2003) and zebrafish (Filby et al., 2010) show HPA axis response to social stressors, and social buffering of stress (Ziv et al., 2012). The current study completes this picture by showing that behavioral effects of HPA dysregulation and their reversal with known antidepressants can already be detected at larval stages. The *gr*^*s357*^ mutant zebrafish may therefore prove useful for evaluating the effect of chronic stress on brain circuitry and also as a convenient model for *in vivo* drug screens in search of novel therapeutic agents.

### Conflict of interest statement

The authors declare that the research was conducted in the absence of any commercial or financial relationships that could be construed as a potential conflict of interest.
